# Single-Source Vapor-Deposited Cs_2_AgBiBr_6_ Thin Films for Lead-Free Perovskite Solar Cells

**DOI:** 10.3390/nano9121760

**Published:** 2019-12-11

**Authors:** Ping Fan, Huan-Xin Peng, Zhuang-Hao Zheng, Zi-Hang Chen, Shi-Jie Tan, Xing-Ye Chen, Yan-Di Luo, Zheng-Hua Su, Jing-Ting Luo, Guang-Xing Liang

**Affiliations:** Shenzhen Key Laboratory of Advanced Thin Films and Applications, College of Physics and Optoelectronic Engineering, Shenzhen University, Shenzhen 518060, China; fanping@szu.edu.cn (P.F.); P2385284535@163.com (H.-X.P.); zhengzh@szu.edu.cn (Z.-H.Z.); chen798491170@126.com (Z.-H.C.); 2017171042@email.szu.edu.cn (S.-J.T.); ymt_0198@163.com (X.-Y.C.); lyandidi@163.com (Y.-D.L.); zhsu@szu.edu.cn (Z.-H.S.); luojt@szu.edu.cn (J.-T.L.)

**Keywords:** lead-free, perovskite solar cell, thin film, single-source vapor deposition

## Abstract

Lead-free double perovskites have been considered as a potential environmentally friendly photovoltaic material for substituting the hybrid lead halide perovskites due to their high stability and nontoxicity. Here, lead-free double perovskite Cs_2_AgBiBr_6_ films are initially fabricated by single-source evaporation deposition under high vacuum condition. X-ray diffraction and scanning electron microscopy characterization show that the high crystallinity, flat, and pinhole-free double perovskite Cs_2_AgBiBr_6_ films were obtained after post-annealing at 300 °C for 15 min. By changing the annealing temperature, annealing time, and film thickness, perovskite Cs_2_AgBiBr_6_ solar cells with planar heterojunction structure of FTO/TiO_2_/Cs_2_AgBiBr_6_/Spiro-OMeTAD/Ag achieve an encouraging power conversion efficiency of 0.70%. Our preliminary work opens a feasible approach for preparing high-quality double perovskite Cs_2_AgBiBr_6_ films wielding considerable potential for photovoltaic application.

## 1. Introduction

Organic–inorganic hybrid lead halide perovskite material family for photovoltaic application has attracted considerable attention in the past few years due to its excellent optoelectronic properties [1,2]. This material has also been rapidly developed with a power conversion efficiency (PCE) exceeding 24% [3]. However, two challenging problems still need to be solved, namely, lead toxicity and long-term stability issues, for the commercial application of perovskite solar cells (PSCs) [4,5]. Therefore, to date, many researchers have given considerable attention to the development of inorganic and/or environmentally friendly Pb-free perovskite materials to obtain highly stable nontoxic PSC devices [6,7]. A commonly applied approach toward Pb-free perovskites is to replace heavy metal Pb^2+^ ions with the divalent metal Sn^2+^ ions. However, the stability of tin-based PSC device under ambient environment is not ideal, because the divalent Sn^2+^ ion is easily oxidized into tetravalent Sn^4+^ ion [8]. Alternatively, nontoxic bismuth (Bi) based inorganic perovskites have been applied in solar cell devices as light absorber layers due to their high stability. However, Bi-based perovskites (A^+1^B^2+^X^−1^_3_) cannot form three-dimensional (3D) structure of traditional perovskites, but only have zero-dimensional (0D) or two-dimensional (2D) structure of A^+1^_3_B^3+^_2_X^−1^_9_, which commonly results in unfavorable optoelectronic properties, such as high exciton binding energy, low carrier mobility, short carrier diffusion length, and high trap-state density [9,10]. Recently, the novel inorganic double perovskite material (Cs_2_AgBiX_6_ (X = Cl, Br)) has drawn research attention due to their high stability and nontoxicity, and their single crystal with highly symmetric cubic structure has been synthesized in succession [11,12,13]. This newly discovered double perovskite, especially the Cs_2_AgBiBr_6_, has been theoretically and experimentally tested to be a potential candidate for photovoltaic application due to their advantages of long carrier recombination lifetime, low toxicity, and high stability [5,14,15,16,17]. Moreover, in recent years, some articles regarding the perovskite Cs_2_AgBiBr_6_ solar cells have been reported [18,19,20,21,22], and its highest PCE achieved is close to 2.51% [23]. The power conversion efficiency (PCE) of Cs_2_AgBiBr_6_ solar cells is much lower than that of conventional perovskite devices, mainly because double perovskite Cs_2_AgBiBr_6_ is the semiconductor material with an indirect bandgap and narrow optical absorption wavelength range (its bandgap is ~2 eV). As a result, the current density of Cs_2_AgBiBr_6_ solar cells is very low and the perovskite efficiency is not high. Further improvement can be obtained by the bandgap engineering of double perovskite. Additionally, its bandgap can be tunable from 0.5 eV to 2.7 eV [24]. For example, element Tl (I) can be added into Cs_2_AgBiBr_6_ to form Cs_2_(Ag_1−a_Bi_1−b_)Tl*_x_*Br_6_ (*x* = 0.075), with a direct bandgap of 1.57 eV [25]. Two methods are available for preparing double perovskite films, namely, solution-processing and vapor deposition. Greul et al. used a simple one-step spin-coating method to prepare the mesostructure Cs_2_AgBiBr_6_ solar cells. The absorber layer was fabricated by directly spin-coating the precursor of BiBr_3_, AgBr, and CsBr on the porous TiO_2_, but the surface morphology of Cs_2_AgBiBr_6_ films appeared to be poor, with high roughness and defect density [21]. Especially, perovskite films with pinholes may cause the direct contact between electron and hole transport layers, which result in photogenerated carrier recombination loss. This is mainly because the perovskite films that are prepared by solution-processing are very sensitive to conditions of film forming, such as annealing temperature [26], solution concentration [27], precursor composition [28], and solvent selection [29]. Gao et al. and Wu et al. obtained flat Cs_2_AgBiBr_6_ films by anti-solvent and low-pressure assisted methods, respectively, but the low solubility (less than 0.6 mol/L) of double perovskite precursors in common solvents limits the preparation of high-quality films, as well as their commercial applications. In addition, Wang et al. prepared Cs_2_AgBiBr_6_ solar cells by the sequential vapor deposition. The fabrication is complicated for sequentially evaporating AgBr, BiBr_3_ and CsBr powder under high vacuum and determining their suitable composition ratio, and the composition ratios of the prepared Cs_2_AgBiBr_6_ films greatly deviated from the ideal stoichiometry, which is not conducive to the preparation of high-efficiency solar cells. Single-source vapor deposition is an alternative method for the preparation of perovskite films [30,31]. The materials to be deposited is placed on a metal heater, and then rapidly evaporated onto the substrates by adjusting the heating current. Through this simple method, the Cs_2_AgBiBr_6_ films have the advantages of good smoothness, high uniformity, and high crystallinity. Inspired by this, we fabricated Cs_2_AgBiBr_6_ solar cells by single-source vapor deposition.

In this work, we successfully fabricated double perovskite Cs_2_AgBiBr_6_ film by single-source vapor deposition. The annealing effects on the crystallinity and optical properties of Cs_2_AgBiBr_6_ films were systematically investigated. We found that the Cs_2_AgBiBr_6_ films annealed at 300 °C for 15 min had better properties with high crystallinity, good uniformity, and free pinholes. Planar heterojunction perovskite solar cells based on this film were prepared. By adjusting the annealing conditions and film thickness, the Cs_2_AgBiBr_6_ solar cell devices show an optimized PCE of approximately 0.70%.

## 2. Materials and Methods 

All of the preparation chemical materials were used without any further purification. All of the fabrication processes were operated under ambient conditions, except the preparation of the hole transporting layer (HTL) and the post-annealing, which were conducted in a glove box filled with nitrogen.

### 2.1. Cs_2_AgBiBr_6_ Crystal and Powder Preparation

The double perovskite Cs_2_AgBiBr_6_ crystals were prepared via the modified crystallization method [5]. The detailed processes are as follows: 426 mg CsBr (2.00 mmol, 99.5%, MACKLIN, Shanghai, China), 188 mg AgBr (1.00 mmol, 99.9%, MACKLIN, Shanghai, China), and 449 mg BiBr_3_ (1.00 mmol, 99%, Alfa Aesar, Ward Hill, MA, USA) powder were sequentially dissolved in 12 mL of hydrobromic acid (HBr, ACS, 48%, Aladdin, Shanghai, China) solution in a transparent glass bottle. Subsequently, the glass bottle was tightly sealed and placed into the petri dish filled with silicone oil. The silicone oil was gradually heated to 110 °C and then held for approximately 3 h to dissolve the raw material entirely and obtain a clear precursor solution. Subsequently, the solution was smoothly cooled down to 58 °C at a rate of 3 °C/h. Thereafter, minute Cs_2_AgBiBr_6_ crystals can be observed. The solution was kept at 58 °C for another 9 h to further promote crystal growth. Subsequently, the solution was cooled down to 35 °C at 1 °C/h. Finally, the well-grown Cs_2_AgBiBr_6_ crystals were collected by filtrating and were then washed by isopropyl alcohol (AR, ≥99.5%, Aladdin, Shanghai, China) three times. After drying in the oven, the prepared crystals could be ground to powder for use.

### 2.2. Device Fabrication

Fluorine-doped tin oxide (FTO) glasses (2.0 × 2.0 cm^2^, Sigma-Aldrich, Saint Louis, MO, USA) were ultrasonically sequentially then cleaned with industrial detergent, deionized water, and ethanol for 30 min. After treatment with ultraviolet (UV) ozone cleaning system for 15 min, the titanium dioxide (TiO_2_) was fabricated by spin-coating TiO_2_ precursor solution [32] at 3000 rpm for 30 s and then annealed at 450 °C for 1 h. Subsequently, the Cs_2_AgBiBr_6_ films were deposited onto the TiO_2_-coated substrates by single-source vapor deposition. First, Cs_2_AgBiBr_6_ powder was loaded into the tungsten boat, and the cleaned FTO substrates were then fixed onto a rotatable holder above the evaporation source. The distance between the substrates and the evaporation source is approximately 20 cm. When the pressure in the vacuum chamber dropped down to 5.0 × 10^−4^ Pa, the evaporation source was smoothly heated to evaporate the perovskite powder by adjusting the heating current from 0 A to 120 A at a rate of 20 A/min. Meanwhile, the substrate holder was rotating at a rate of 20 rpm. The Cs_2_AgBiBr_6_ powder was completely evaporated in only a few minutes, and the Cs_2_AgBiBr_6_ films were then formed. The device performance of PSCs was optimized by varying the post-annealing temperatures (150 °C, 250 °C, 300 °C, and 350 °C), annealing times (5 min, 15 min, 30 min, and 65 min) and film thicknesses (167 nm, 238 nm, and 297 nm). Thereafter, the HTL was prepared by spin-coating 2,2′,7,7′-tetrakis-(*N*,*N*-di-4-methoxyphenylamino)-9,9′-spirobi-fluorene (Spiro-OMeTAD, 99.7%, MACKLIN, Shanghai, China) solution on the perovskite film at 3000 rpm for 30 s. The preparation processes of the Spiro-OMeTAD solution are as follows. First, 145 mg Spiro-OMeTAD powder was dissolved in 2 mL of chlorobenzene (99.9%, Sigma-Aldrich, Saint Louis, MO, USA). Second, 57 μL of 4-*tert*-butylpyridine (AR, Sigma-Aldrich, Saint Louis, MO, USA) and 35 μL of bis(trifluoromethane)sulfonamide lithium salt (99.9%, MACKLIN, Shanghai, China) solution (520 mg/mL) in acetonitrile (super dehydrated, Wako, Tokyo, Japan) were sequentially added to the Spiro-OMeTAD solution. Third, the solution was filtered. Finally, approximately 80-nm-thick Ag thin film was deposited on the top of the device at a rate of 0.5 Å/s by the vacuum thermal evaporation method.

### 2.3. Characterization

The crystallinity of the samples was analyzed by X-ray diffraction (XRD, Ultima IV, Rigaku, Tokyo, Japan) by using CuKα radiation (λ = 0.15406 nm) operated at 40 kV and 40 mA. The scanning electron microscopy (SEM) images were measured by SUPRA 55 Sapphire SEM (Zeiss, Oberkochen, Germany) with an accelerated voltage of 3 kV. UV–visible (UV–vis) absorption spectra were measured by a UV–vis–near-infrared spectrophotometer (Lambda 950, Perkin Elmer, Akron, OH, USA) with a wavelength range of 175–3300 nm and resolution of <0.05 nm. Photoluminescence (PL) spectra of the products were measured by a Raman spectrometer (inVia, Renishaw, Gloucestershire, England), with an excited wavelength of 532 nm (50 mW) and a spectral resolution of 1 cm^−1^. The film thicknesses of perovskite films were measured on the DEKTAKXT profilometer (Bruker, Billerica, MA, USA). The femtosecond transient absorption (TA) spectra were recorded with the multimodal ultrafast spectroscopy system (SOLSTICS-1K, Newport Corporation, Irvine, CA, USA) with a 35 femtosecond Ti: sapphire chirped pulse amplifier (Spectra-Physics Spitfire Pro 35) operating at a 1 kHz repetition rate and generating 35 fs pulse that was centered at approximately 800 nm. The valence band (VB) of the perovskite material in this work was performed by UV photoelectron spectroscopy (UPS). The spectrometer setup is equipped with a monochromatic He I source (21.2 eV) and a VG Scienta R4000 analyzer (Uppsala, Sweden). The current density–voltage (J–V) curves of the PSCs were measured by a Keithley 2400 (SolarIV-150A, Zolix, Beijing, China) under simulated AM 1.5 Solar Simulator (100 mW/cm^2^). The active area of the solar cell was approximately 0.1 cm^2^. The external quantum efficiency (EQE) was measured while using an EQE 200 Oriel integrated system (SCS1011, Zolix, Beijing, China) under 1.5 AM white light.

## 3. Results and Discussion

### 3.1. Crystalline Properties of Cs_2_AgBiBr_6_ Films

**Figure 1:**
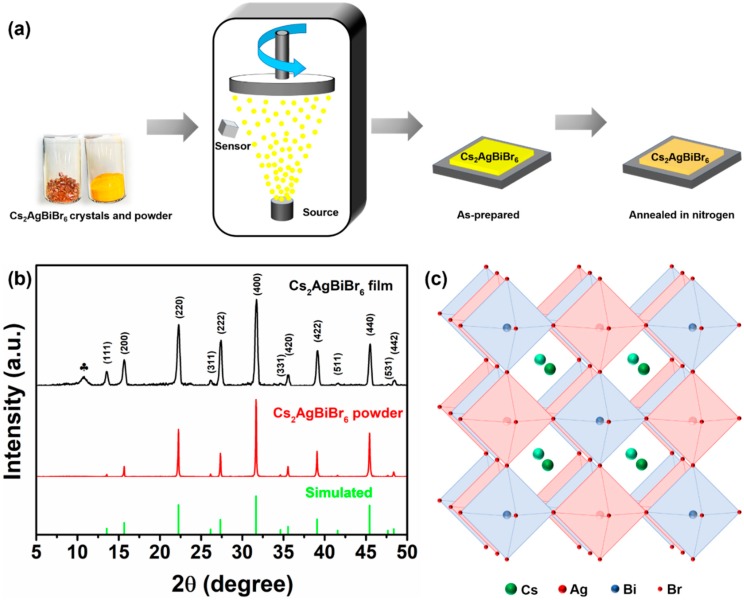
Schematic of Cs_2_AgBiBr_6_ film preparation (**a**). X-ray diffraction (XRD) patterns of Cs_2_AgBiBr_6_ film and powder (**b**). Crystal structure of Cs_2_AgBiBr_6_ (**c**). The position of reflection labeled by club (♣) indicates the additional phase of BiOBr.

As shown in Figure 1a, the single-source vapor deposition method was used for preparing our Cs_2_AgBiBr_6_ films. High-quality Cs_2_AgBiBr_6_ single crystals were initially produced according to our modified method, and the detailed process is shown in Experimental Details (Supporting Information). Our prepared Cs_2_AgBiBr_6_ single crystals show an octahedral structure with bright surfaces and a size up to a millimeter (Figure S1) [5,18,20]. Our prepared Cs_2_AgBiBr_6_ powder could endure high heating temperature up to 430 °C without weight loss (Figure S2). The as-prepared Cs_2_AgBiBr_6_ films are yellow in comparison with orange Cs_2_AgBiBr_6_ powder. The films turned into orange after being thermally annealed at high temperature.

As shown in Figure 1b, the XRD patterns of Cs_2_AgBiBr_6_ film and powder were in agreement with the simulated result. The film diffraction peaks located at 13.6°, 15.7°, 22.2°, 26.1°, 27.3°, 31.7°, 34.6°, 35.6°, 39.1°, 41.6°, 45.4°, 47.7°, and 48.4° could be indexed as the (111), (200), (220), (311), (222), (400), (331), (420), (422), (511), (440), (531), and (442) planes of Cs_2_AgBiBr_6_ perovskite, respectively [5,13]. This phenomenon indicates that our method synthesized the high-crystallinity Cs_2_AgBiBr_6_ films. Figure 1c shows the crystal structure of perovskite Cs_2_AgBiBr_6_. Moreover, the additional minor diffraction peak located at 10.82° represents the BiOBr side phase, which indicates that the perovskite Cs_2_AgBiBr_6_ partially decomposed into BiBr_3_ during deposition and then hydrolyzed into BiOBr during post-annealing.

**Figure 2:**
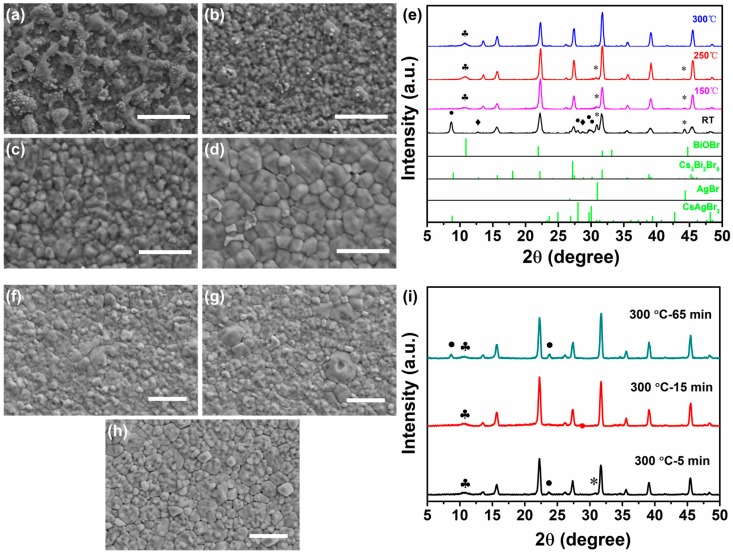
SEM images and XRD patterns of Cs_2_AgBiBr_6_ films annealed at different temperatures for different time. (**a**–**d**) Cs_2_AgBiBr_6_ films were annealed at different temperatures for 30 min respectively. RT (ca. 25 °C) (**a**), 150 °C (**b**), 250 °C (**c**), and 300 °C (**d**). (**e**) XRD patterns of Cs_2_AgBiBr_6_ films without and with annealing at 150, 250 and 300 °C. (**f**–**h**) Cs_2_AgBiBr_6_ films were annealed at 300 °C for different time. 5 min (**f**), 15 min (**g**), 65 min (**h**). (**i**) XRD patterns of Cs_2_AgBiBr_6_ films annealed at 300 °C for 5, 15 and 65 min, respectively. The positions of reflections labeled by circle (•), diamond (♦), asterisk (*), and club (♣) indicate the additional phases of CsAgBr_2_ (PDF#38-0850), Cs_3_Bi_2_Br_9_ (PDF#44-0714), AgBr (PDF#06-0438), and BiOBr (PDF#52-0084), respectively. The scale bars in the SEM images are all 1 μm.

The samples were thermally annealed at different temperatures to investigate the effect of post-annealing on the surface morphology and crystalline of Cs_2_AgBiBr_6_ film. The as-prepared Cs_2_AgBiBr_6_ films were respectively annealed at 150 °C, 250 °C and 300 °C for 30 min in the nitrogen-filled glove box. Figure 2a–d shows the SEM surface morphology of Cs_2_AgBiBr_6_ films with and without annealing. It can be observed that the as-prepared Cs_2_AgBiBr_6_ film is covered completely, but with many island masses. However, the Cs_2_AgBiBr_6_ films became remarkably uniform and smooth after thermally annealing. Besides, grain size gradually increased up to several hundreds of nanometers with the annealing temperature increasing from 150 °C to 300 °C, indicating higher crystalline Cs_2_AgBiBr_6_ films that were obtained by thermally annealing. Nevertheless, for the film annealed at 350 °C, the film coverage became very poor, as shown in Figure S3. It can be inferred that the Cs_2_AgBiBr_6_ film may partially decompose under much high annealing temperature (350 °C), which was confirmed by the following XRD results. The XRD patterns of the Cs_2_AgBiBr_6_ films with and without annealing are shown in Figure 2e. The as-prepared Cs_2_AgBiBr_6_ films feature additional diffraction peaks at 8.70°, 12.76°, 28.02°, 28.74°, 29.72°, 30.10°, 30.86°, and 44.20°, which indicates the unexpected phases of CsAgBr_2_, Cs_3_Bi_2_Br_9_, and AgBr [15,21]. This XRD results imply that the double perovskite Cs_2_AgBiBr_6_ decomposed into CsAgBr_2_, Cs_3_Bi_2_Br_9_, and AgBr during the process of deposition. Notably, the side phases gradually decreased with the treatment of post annealing from 150 °C to 300 °C. Additionally, high-purity Cs_2_AgBiBr_6_ films without the additional phases of CsAgBr_2_, Cs_3_Bi_2_Br_9_ and AgBr were obtained under the annealing temperature of 300 °C. However, for the film annealed at 350 °C, it is obvious that we could see the diffraction peaks of side phases CsAgBr_2_ and Cs_3_Bi_2_Br_9_, being labeled by circle (•) and diamond (♦), respectively, in Figure S4. Their peak intensity is much stronger than that of Cs_2_AgBiBr_6_, suggesting that Cs_2_AgBiBr_6_ film would decompose into CsAgBr_2_ and Cs_3_Bi_2_Br_9_ when the annealing temperature was up to 350 °C. However, there is no decomposition in Cs_2_AgBiBr_6_ crystals in the range of room temperature to 350 °C, according to the literature that was reported by Gao et al. [20], indicating that high-quality Cs_2_AgBiBr_6_ crystals may feature higher thermal decomposition temperature than Cs_2_AgBiBr_6_ films. In addition, the club (♣) indicates the diffraction peaks from the side phase BiOBr, as discussed in Figure 1b. From the XRD patterns of Cs_2_AgBiBr_6_ films, it is found that there are three major diffraction peaks of (220), (400), and (440) planes. Their corresponding peak intensity, as a function of annealing temperature, is displayed in Figure S5, respectively. We find that the intensity of the three major peaks increased after annealing and performed the most strongly between the annealing temperatures of 250 °C and 300 °C, which indicated that Cs_2_AgBiBr_6_ films with higher crystalline were obtained with post-annealing treatment. In order to further optimize the film quality, our as-prepared Cs_2_AgBiBr_6_ films were also annealed at 300 °C for 5 min, 15 min, and 65 min, respectively. The SEM images show that the films from 5 min, 15 min, and 30 min are all dense and uniform, whereas Figure 2f–h show the films from 65 min have many defects, such as pinholes and cracks. The XRD patterns of Cs_2_AgBiBr_6_ films annealed at 300 °C for 5 min, 15 min and 65 min are displayed in Figure 2i. The diffraction peak intensity from the preferred orientations of (220), (400) and (440) planes as a function of annealing time was carried out in Figure S6. It could be observed that the peak intensity of (220), (400), and (440) planes increased with the annealing time increasing from 5 min to 30 min, whereas it decreased with more annealing time of 65 min. So suitable annealing time is beneficial to obtain the high crystalline Cs_2_AgBiBr_6_ films. Moreover, the diffraction peaks of Cs_2_AgBiBr_6_ films from 5 min, 30 min and 65 min, labeled with circle (•), asterisk (*), and club (♣), correspondingly indicate the additional phases of CsAgBr_2_, AgBr, and BiOBr, which suggests that the annealing time of 15 min can sufficiently obtain the desired Cs_2_AgBiBr_6_ films.

### 3.2. Photophysical Properties and Energy Band Structure of Cs_2_AgBiBr_6_ Films

**Figure 3:**
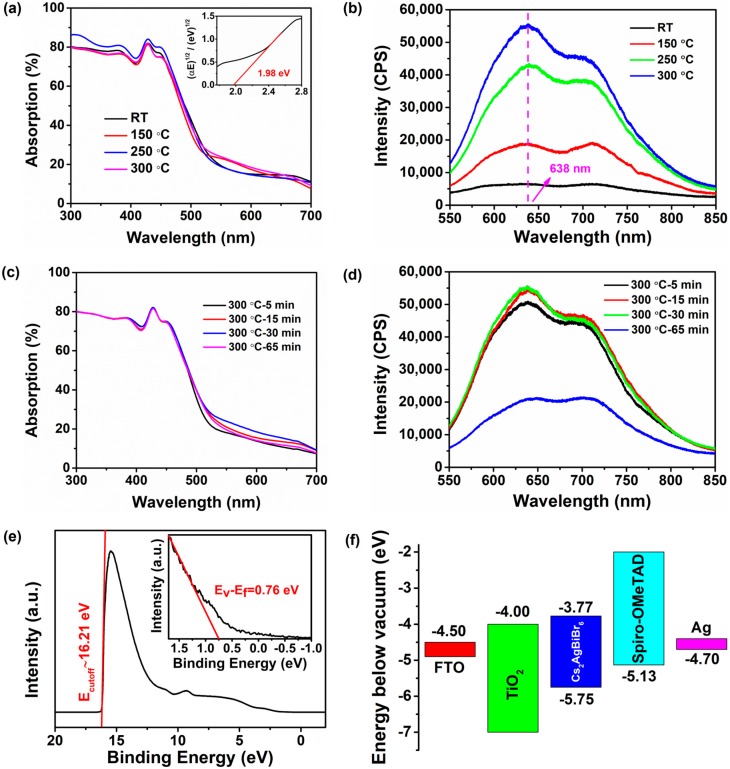
(**a**,**b**) UV-vis and photoluminescence (PL) spectra of Cs_2_AgBiBr_6_ films with and without annealing. As-prepared Cs_2_AgBiBr_6_ films were annealed at 150 °C, 250 °C and 300 °C for 30 min respectively. Inset in (**a**) is the Tuat plot obtained from the optical absorption spectrum. (**c**,**d**) UV-vis and PL spectra of Cs_2_AgBiBr_6_ films annealed at 300 °C for 5 min, 15 min, 30 min, and 65 min respectively. (**e**) UPS spectra of Cs_2_AgBiBr_6_ film annealed at 300 °C for 15 min. (**f**) Energy band diagram of Cs_2_AgBiBr_6_ solar cell with the planar structure of FTO/compact TiO_2_/Cs_2_AgBiBr_6_/Spiro-OMeTAD/Ag.

The UV–vis absorption and PL spectra of Cs_2_AgBiBr_6_ films annealed at different temperatures were also measured in order to further investigate the optical properties of Cs_2_AgBiBr_6_ films, as shown in Figure 3a,b. The as-prepared Cs_2_AgBiBr_6_ films were annealed at 150 °C, 250 °C, and 300 °C for 30 min respectively in the nitrogen-filled glove box. It can be observed that all the absorption spectra feature three parts, namely, a smooth absorption of approximately 80% below 450 nm, a steep absorption in the wavelength region of 450–520 nm, and a weak absorption lower than 25% above 520 nm, which indicates that double perovskite Cs_2_AgBiBr_6_ film has high thermal stability even under 300 °C, whereas the typical perovskites decompose at 150 °C. In the literatures previously reported, there is commonly a sharp absorption peak located at ~450 nm that might arise from a direct bismuth s-p transition [33], whereas no such feature can be observed in our Cs_2_AgBiBr_6_ film. That probably attributes to our different methods for the preparation of perovskite thin films. The Tauc plot (see inset in Figure 3a), which was obtained from the absorption spectrum of Cs_2_AgBiBr_6_ film, determined an indirect band gap of approximately 1.98 eV, which is in agreement with the previously reported results [21]. From the PL spectra of Cs_2_AgBiBr_6_ films with different annealing temperatures (Figure 3b), we found that only two thin films annealed at 250 °C and 300 °C feature a wide typical PL peak located at approximately 638 nm (1.94 eV) and the PL peak of the thin film annealed at 150 °C is not obvious, which indicated that the post-annealing process with more than annealing temperature of 250 °C is necessary for obtaining desired Cs_2_AgBiBr_6_ film. It is worth noting that the PL spectra of our Cs_2_AgBiBr_6_ films exhibit an additional peak at ~710 nm (~1.75 eV), which is similar to the situation in the previous literature [20]. The report pointed out that this additional peak originates from the photon-assisted indirect band transitions processes. However, no phonon-assisted processes with transitions at ~1.75 eV could be observed in our Tauc plot, which suggests that this additional peak might arise from the direct bandgap emission of Cs_2_AgBiBr_6_. Besides, the peak intensity became stronger with the annealing temperature increasing. For comparison, the PL spectrum of our synthesized Cs_2_AgBiBr_6_ crystal was also measured, as shown in Figure S7. The measured result matched well with that of Cs_2_AgBiBr_6_ film annealed at 300 °C, which suggests that high-homogeneity Cs_2_AgBiBr_6_ films were obtained with the treatment of annealing. The optical properties of Cs_2_AgBiBr_6_ films with different annealing time (5 min, 15 min, 30 min, and 65 min) were also studied (Figure 3c,d). It can be seen that the optical absorption with different annealing time is very similar to each other in the whole wavelength range (Figure 3c), as discussed above, although the Cs_2_AgBiBr_6_ films annealed at 300 °C for 65 min have many pinholes and cracks shown in the SEM image. As shown in Figure 3d, all of the PL spectra of Cs_2_AgBiBr_6_ films annealed at 300 °C with different annealing time possessed similar obvious PL peaks, except the thin film annealed at 300 °C for 65 min. Additionally, the PL peak intensity increased with the annealing time increasing from 5 min to 15 min, but it hardly changed when the annealing time was up to 30 min. The increased PL peak intensity indicates that non-radiative decay in our Cs_2_AgBiBr_6_ films is significantly suppressed and. as a result, the number of defects decrease by our annealing [34]. These results imply that too much annealing time under high temperature is not favorable for the preparation of high-quality Cs_2_AgBiBr_6_ film and, in our experiment, the annealing time of 30 min is suitable for obtaining the desire thin film. 

UPS was used to calculate the energy band structure of the annealed Cs_2_AgBiBr_6_ film as shown in Figure 3e,f. The work function (*E_f_*) can be calculated by the equation *E_f_* = 21.2 eV (He I) − *E*_cutoff_, where *E*_cutoff_ is 16.21 eV, as presented in Figure 3e, and the resulting value of *E*_F_ is 4.99 eV. The linear extrapolation in the low binding-energy region (see inset in Figure 3e) indicates the value of (*E*_V_ − *E*_F_), leading to an *E*_V_ of 5.75 eV. As a result, the conduction band (*E*_C_) energy of Cs_2_AgBiBr_6_ film can be estimated by the value of (*E*_V_ − *E*_g_) and the relative *E*_C_ value is 3.77 eV. The *E*_F_ energy level is near the top of VB (*E*_V_), which suggests that the prepared Cs_2_AgBiBr_6_ film is probably a p-type semiconductor. We carried out the energy band diagram of Cs_2_AgBiBr_6_ solar cell with the planar structure of FTO/TiO_2_/Cs_2_AgBiBr_6_/Spiro-OMeTAD/Ag, based on the calculating results of energy band structure of Cs_2_AgBiBr_6_ film, as shown in Figure 3f. According to the energy band theory, the photogenerated electrons and holes in the Cs_2_AgBiBr_6_ film can separately transport through the hole and electron blocking layers (TiO_2_ and Spiro-OMeTAD) to the electrodes (FTO and Ag), which would meet the requirement for the preparation of Cs_2_AgBiBr_6_ solar cell.

### 3.3. Photogenerated Carrier Lifetime of Cs_2_AgBiBr_6_ Films

**Figure 4:**
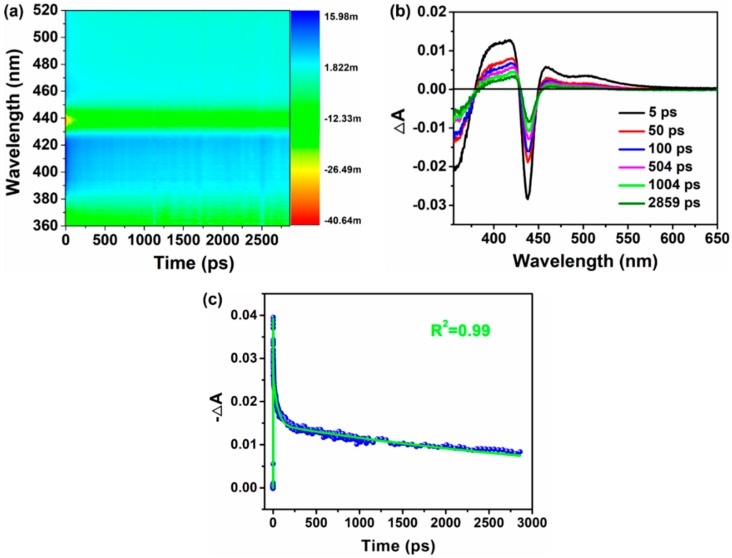
Femtosecond transient absorption (TA) spectra of Cs_2_AgBiBr_6_ film with an excited energy of 350 nm. (**a**) Pseudocolor TA plot. (**b**) TA spectra at indicated delay time from 5 ps to 2859 ps. (**c**) Ground-state bleach (GSB) decay dynamic probed at 439 nm. The as-prepared Cs_2_AgBiBr_6_ films were annealed at 300 °C for 15 min.

The femtosecond TA spectra of Cs_2_AgBiBr_6_ film were carried out to estimate the lifetime of photoinduced charge carriers of Cs_2_AgBiBr_6_ film, as shown in Figure 4. A GSB at 439 nm can be observed for perovskite Cs_2_AgBiBr_6_ film (Figure 4a), which originates from the state-filling effect [35,36]. Two broad photoinduced absorption bands correspondingly centered at 458 nm and 504 nm can be seen at early times and then rapidly turn into a strong bleaching at a long time (Figure 4b). As previously reported [37,38], this characteristic is a typical feature assigned to exciton–exciton interaction. Three components can fit the GSB decay probed at 439 nm, namely, a short-lived lifetime of~1.3 ps, a middle-lived lifetime of ~50 ps, and a long-lived lifetime of ~4.3 ns (Figure 4c). According to the literature [39], the short- and middle-lived components correspond to the subband gap trapping processes, whereas the long-lived component can be attributed to the carrier recombination processes. The much lower carrier recombination lifetime of the Cs_2_AgBiBr_6_ films than that reported by Yang et al. [23] may be ascribed to the higher defect state density that results from the additional phase of BiOBr in our films. Moreover, in the literature by Slavney et al. [5], the Cs_2_AgBiBr_6_ crystals and powder both show a long carrier recombination lifetime of ~660 ns, which is much longer than those from Cs_2_AgBiBr_6_ films, indicating that Cs_2_AgBiBr_6_ films are likely to have more defects than crystals or powder. 

### 3.4. Photovoltaic Performance

**Figure 5:**
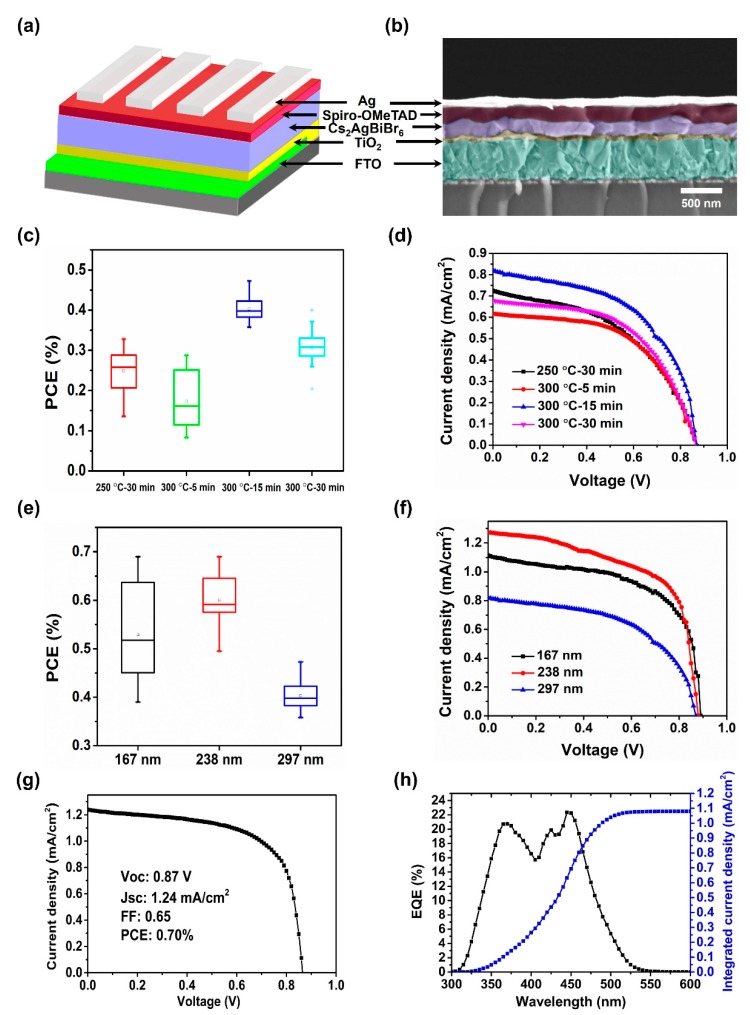
(**a**) Cs_2_AgBiBr_6_ solar cell structure, FTO/compact TiO_2_/Cs_2_AgBiBr_6_/Spiro-OMeTAD/Ag. (**b**) Cross-section SEM image of solar cell device. (**c**) Device performance as a function of annealing temperature and time. (**d**) *J–V* curves of solar cell devices with different annealing temperatures and time. (**e**) Device performance as a function of film thickness. (**f**) *J–V* curves of solar cell devices with different thicknesses of absorber layer. Cs_2_AgBiBr_6_ films were annealed at 300 °C for 15 min. (**g**,**h**) *J–V* curve, and the corresponding EQE spectrum (black) and its integrated current density (blue) of the best performing device.

We fabricated the solar cells based on the planar heterojunction structure of FTO/TiO_2_/Cs_2_AgBiBr_6_/Spiro-OMeTAD/Ag to test the device performance of Cs_2_AgBiBr_6_ film. The Cs_2_AgBiBr_6_ solar cell structure and the cross-section SEM image of solar cell device are shown in Figure 5a,b, respectively. The incorporated Cs_2_AgBiBr_6_ film was annealed at 300 °C for 15 min and its measured thickness is approximately 167 nm. It can be observed that the Cs_2_AgBiBr_6_ grains are comparable with the film thickness, which indicates that most of the photogenerated charges can reach the electron and hole transport layer (TiO_2_ and Spiro-OMeTAD) without encountering grain boundaries, which leads to the reduction of photogenerated carrier recombination loss. This condition is beneficial in the preparation of efficient PSCs. Figure 5c shows the PCEs of Cs_2_AgBiBr_6_ solar cells with different annealing temperatures and time and the corresponding device parameters were summarized in Table S1. A PCE maximum was achieved from the solar cell device assembled with Cs_2_AgBiBr_6_ film annealed at 300 °C for 15 min, which is mainly attributed to the increased *J_sc_* (Figure 5d and Figure S8), caused by the higher crystalline and larger grains of Cs_2_AgBiBr_6_ films after annealing. The improved crystallization and large grains are favorable in reducing the grain boundaries and trapping states, which leads to less recombination loss in Cs_2_AgBiBr_6_ films and longer lifetime of photogenerated carriers. To further optimize the device performance, we also fabricated series of solar cells assembled with different Cs_2_AgBiBr_6_ film thicknesses. Figure 5e shows the device performance of Cs_2_AgBiBr_6_ solar cells as a function of film thickness (167 nm, 238 nm, and 297 nm) and the solar cell parameters are summarized in Table S2. The best PCEs were achieved from a thicker thin film of 238 nm with increased *J_sc_* (Figure 5f and Figure S9). However, the devices that were assembled with extremely thick perovskite films exhibited very low *J_sc_,* which may suffer from the additional phase of BiOBr in our films. According to the literature by Greul et al., solar cells with phase-pure double perovskite films have much higher *J_sc_* than those with side phases of Cs_3_Bi_2_Br_9_ and AgBr. Additionally, the short carrier recombination lifetime in our Cs_2_AgBiBr_6_ films is another reason why our solar cell efficiency is very low. As discussed in Figure 4, our Cs_2_AgBiBr_6_ films has much lower carrier recombination lifetime of ~4.3 ns than those (220 ns and 117 ns) from Greul’s and Wang’s. In addition, our prepared devices show a wide deviation of PCE in the case of 167 nm film when compared to 238 nm and 297 nm cases, which originates from the pinholes in perovskite films (Figure S10). The achieved PCE of the champion device is approximately 0.70% with *V_oc_* of 0.87 V, *J_sc_* of 1.24 mA/cm^2^, and *FF* of 0.65 through the optimized preparation conditions (Figure 5g). The J-V curves of our Cs_2_AgBiBr_6_ solar cells show a hysteresis under forward and backward scanning direction (Figure S11) that can be attributed to the low activation barrier of halide anions during migration in perovskite devices [40]. The integrated current of 1.08 mA/cm^2^ confirmed the tested current density (*J_sc_*) of solar cell (Figure 5h).

## 4. Conclusions

In summary, we demonstrate the double perovskite solar cells with Cs_2_AgBiBr_6_ films that were initially prepared by single-source evaporation deposition. Further crystallized after post-annealing under high temperature, the Cs_2_AgBiBr_6_ films have the advantages of high crystallinity, good smoothness, and free pinholes. By incorporating Cs_2_AgBiBr_6_ films with suitable annealing conditions and film thickness, the solar cell devices represent an optimal PCE of 0.70%. The photovoltaic performance of this perovskite material is expected to be further enhanced by optimizing the energy alignment of charge transporting materials to reduce the energy loss from the interface between perovskite and charge transporting materials. In addition, developing direct bandgap double perovskites by doping can also improve solar cell efficiency. The solar cell devices based on high-quality Cs_2_AgBiBr_6_ films that were prepared by single-source evaporation deposition suggest that this perovskite material can be a potential candidate for environmentally friendly photovoltaic application.

## Supplementary Materials

The following are available online at https://www.mdpi.com/2079-4991/9/12/1760/s1, Figure S1: SEM image of Cs_2_AgBiBr_6_ crystal with typical octahedral morphology; Figure S2: Thermogravimetric analysis of Cs_2_AgBiBr_6_ powder; Figure S3: SEM surface morphology of Cs_2_AgBiBr_6_ film thermally annealed at 350 °C for 30 min; Figure S4: XRD pattern of Cs_2_AgBiBr_6_ film thermally annealed at 350 °C for 30 min. The positions of reflections labeled by circle (•) and diamond (♦) indicate the additional phases of CsAgBr_2_ and Cs_3_Bi_2_Br_9_ respectively; Figure S5: The diffraction peak intensity of (220), (400) and (440) planes of Cs_2_AgBiBr_6_ films as a function of annealing temperature, respectively; Figure S6: The diffraction peak intensity of (220), (400) and (440) planes of Cs_2_AgBiBr_6_ films as a function of annealing time, respectively; Figure S7: Steady-state photoluminescence spectrum of Cs_2_AgBiBr_6_ crystal; Table S1: Device performance of Cs_2_AgBiBr_6_ films with different annealing time and temperatures; Figure S8: The statistical box charts of open-circuit voltage (*V_oc_*), short-circuit current density (J_sc_) and fill factor (FF) of solar cells assembled with Cs_2_AgBiBr_6_ films (297 nm) annealed at 250 °C and 300 °C for different times respectively. The values were obtained from 16 individual devices per annealing condition; Table S2: Parameters of solar cell devices with different Cs_2_AgBiBr_6_ film thicknesses; Figure S9: The statistical box charts of open-circuit voltage (*V_oc_*), short-circuit current density (*J_sc_*) and fill factor (*FF*) of solar cells based on Cs_2_AgBiBr_6_ films with various thin film thickness. The values were obtained from 16 individual devices per annealing condition; Figure S10: SEM surface morphology of Cs_2_AgBiBr_6_ film annealed at 300 °C for 15 min. The film thickness is approximately 167 nm. The areas marked by yellow circles indicate pinholes in the Cs_2_AgBiBr_6_ film. Figure S11. J-V curves of Cs_2_AgBiBr_6_ solar cell, measured by backward scan and forward scan. The Cs_2_AgBiBr_6_ film was prepared at 300°C for 30 min.
