# Supplementary material for: Single-Source Vapor-Deposited Cs2AgBiBr6 Thin Films for Lead-Free Perovskite Solar Cells
Source: Nanomaterials (Basel). 2019 Dec 11;9(12):1760. doi: 10.3390/nano9121760 (PMC6956276; doi:10.3390/nano9121760)
Supplement: Supplementary file 1 [file nanomaterials-09-01760-s001.pdf]

Supporting Information

Single-Source Vapor-Deposited $Cs_2AgBiBr_6$ Thin Films for Lead-Free Perovskite Solar Cells

Ping Fan, Huan-Xin Peng, Zhuang-Hao Zheng, Zi-Hang Chen, Shi-Jie Tan, Xing-Ye Chen, Yan-Di Luo, Zheng-Hua Su, Jing-Ting Luo, and Guang-Xing Liang *

Shenzhen Key Laboratory of Advanced Thin Films and Applications, College of Physics and Optoelectronic Engineering, Shenzhen University, Shenzhen 518060, China; fanping@szu.edu.cn (P.F.); P2385284535@163.com (H.-X.P.); zhengzh@szu.edu.cn (Z.-H.Z.); chen798491170@126.com (Z.-H.C.); 2017171042@email.szu.edu.cn (S.-J.T.); ymt_0198@163.com (X.-Y.C.); lyandidi@163.com (Y.-D.L.); zhsu@szu.edu.cn (Z.-H.S.); luojt@szu.edu.cn (J.-T.L.)

* Correspondence: lgx@szu.edu.cn

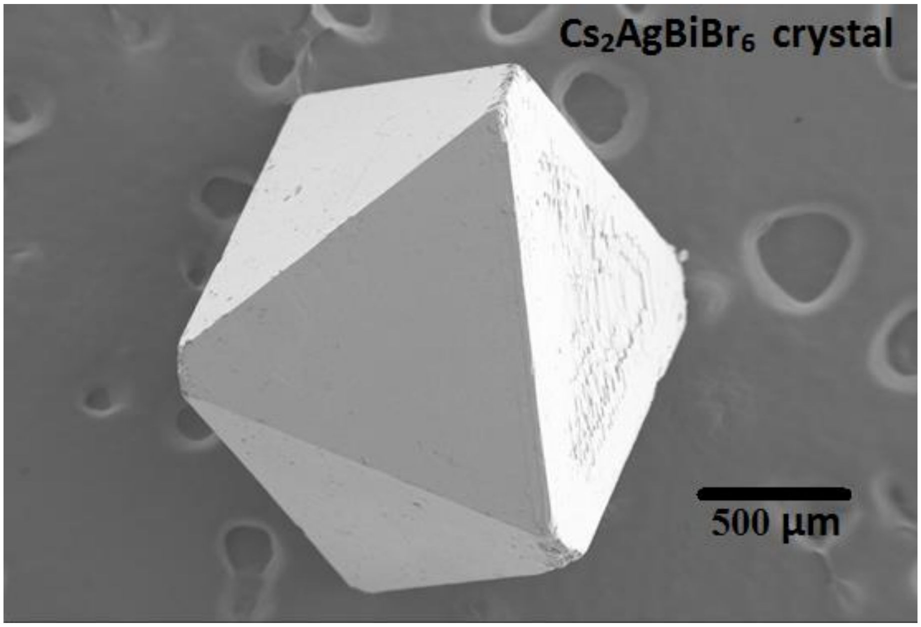

Fig. S1. SEM image of $Cs_2AgBiBr_6$ crystal with typical octahedral morphology.

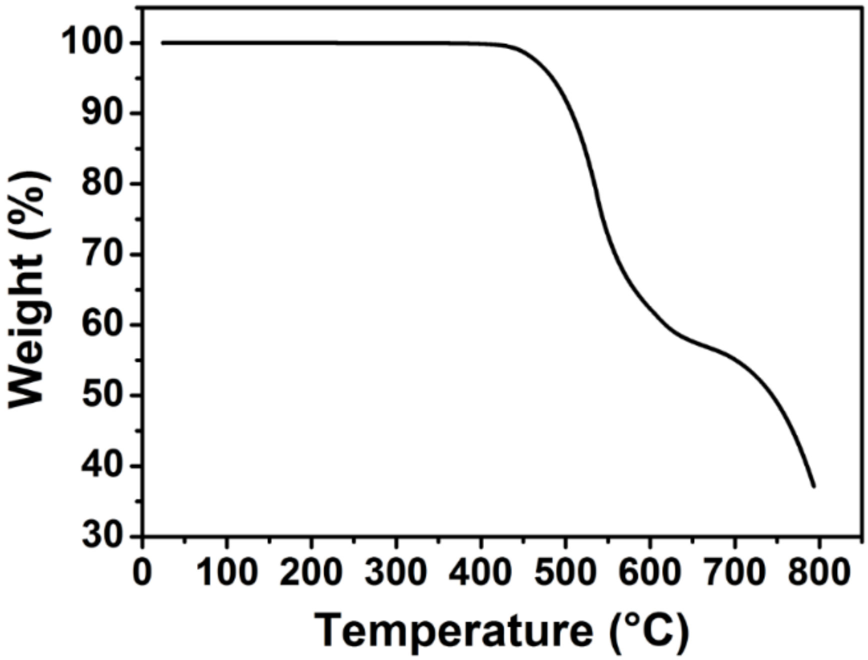

Fig. S2. Thermogravimetric analysis of $Cs_2AgBiBr_6$ powder.

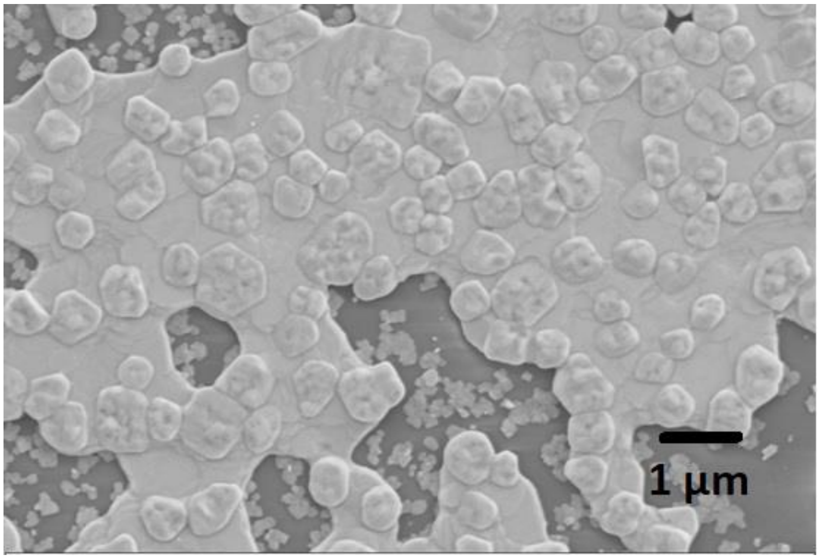

Fig. S3. SEM surface morphology of $Cs_2AgBiBr_6$ film thermally annealed at 350 ℃ for 30 min.

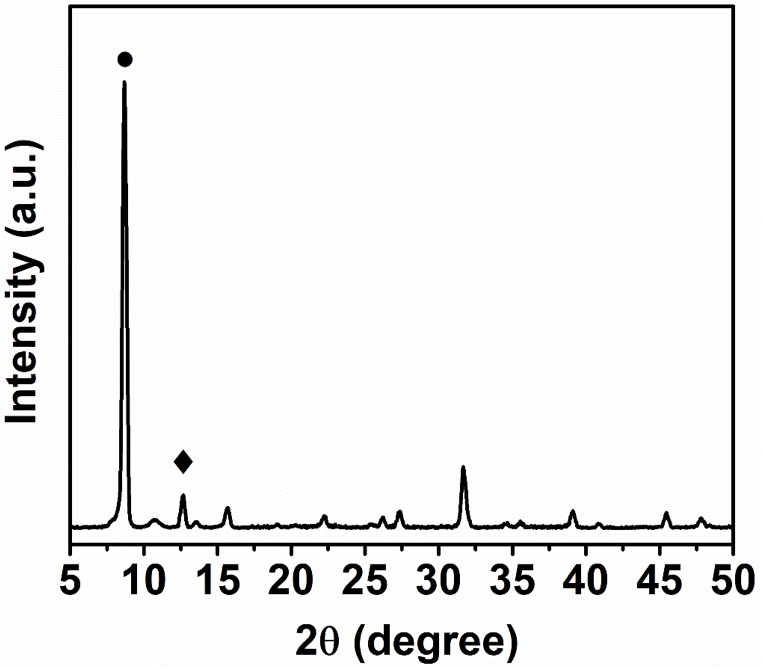

Fig. S4. XRD pattern of $Cs_2AgBiBr_6$ film thermally annealed at 350 ℃ for 30 min. The positions of reflections labeled by circle (•) and diamond (♦) indicate the additional phases of $CsAgBr_2$ and $Cs_3Bi_2Br_9$ respectively.

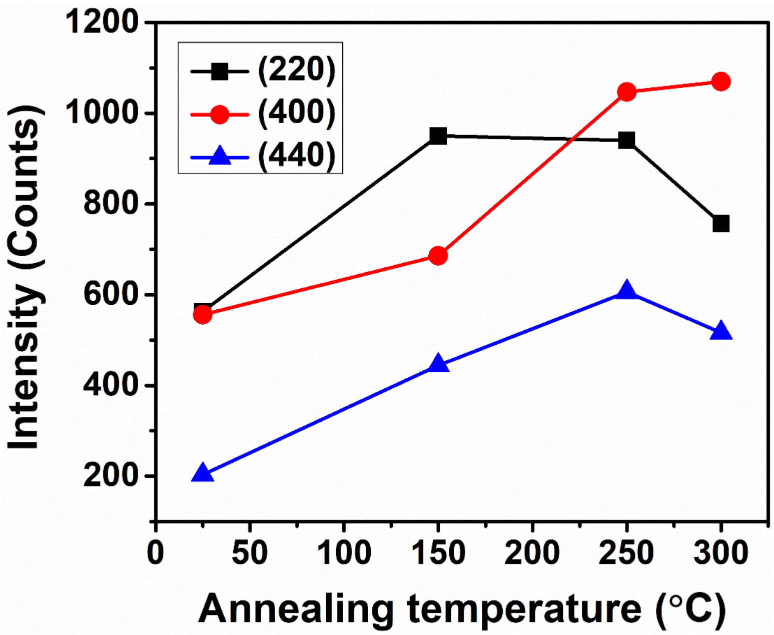

Fig. S5. The diffraction peak intensity of (220), (400) and (440) planes of $Cs_2AgBiBr_6$ films as a function of annealing temperature, respectively.

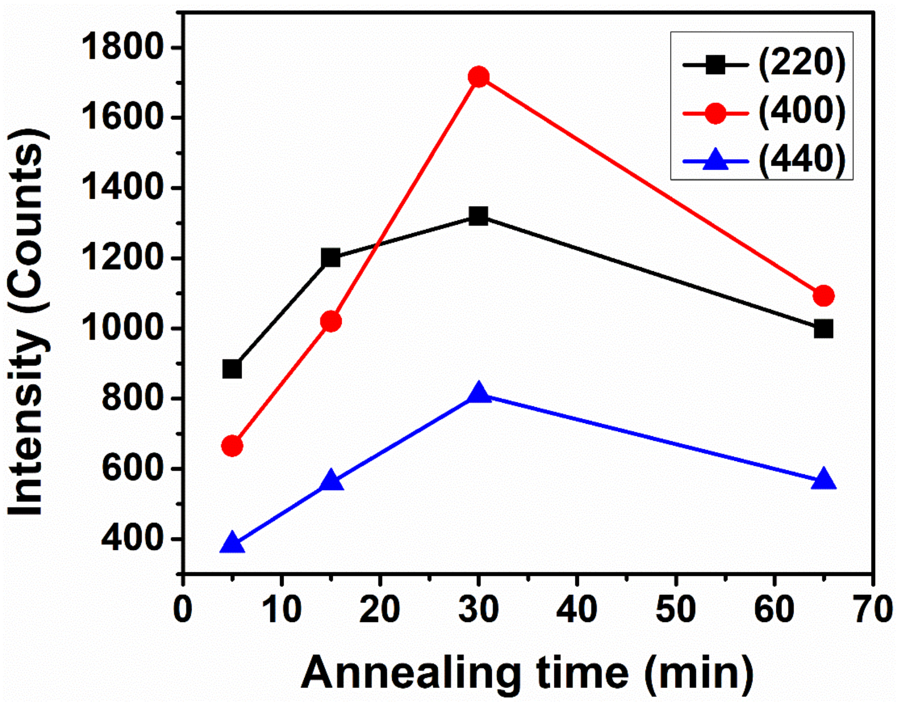

Fig. S6. The diffraction peak intensity of (220), (400) and (440) planes of $Cs_2AgBiBr_6$ films as a function of annealing time, respectively.

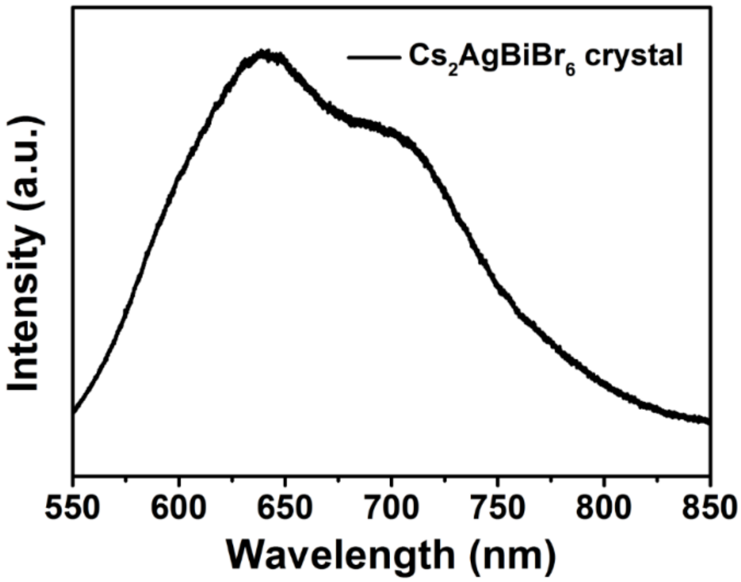

Fig. S7. Steady-state photoluminescence spectrum of $Cs_2AgBiBr_6$ crystal.

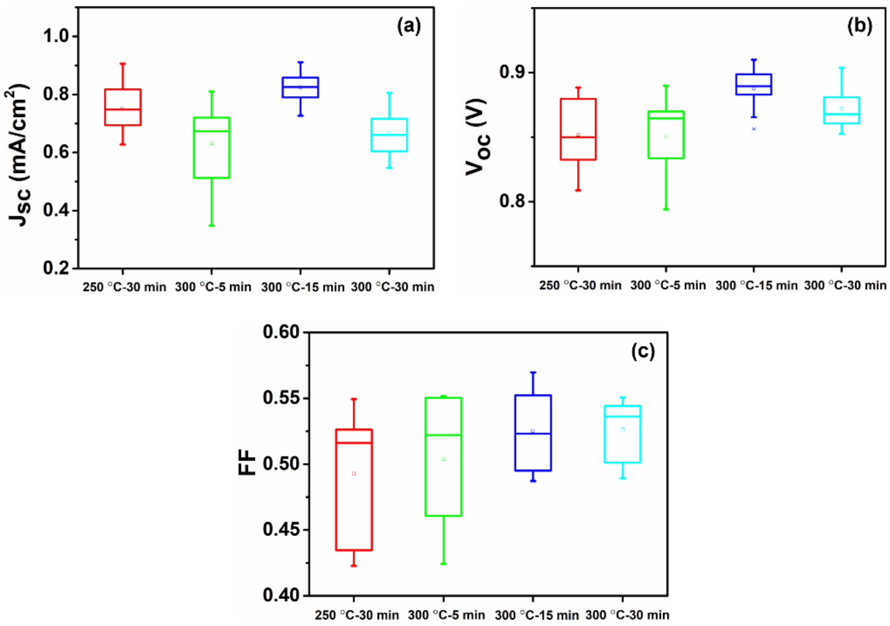

Fig. S8. The statistical box charts of open-circuit voltage (V_{oc}), short-circuit current density (J_{sc}) and fill factor (FF) of solar cells assembled with $Cs_2AgBiBr_6$ films (297 nm) annealed at 250 ℃ and 300 ℃ for different times respectively. The values were obtained from 16 individual devices per annealing condition.

Table S1. Device performance of $Cs_2AgBiBr_6$ films with different annealing time and temperatures.

Sample	Jsc (mA/cm²)	Voc (V)	FF	PCE (%)
$Cs_2AgBiBr_6$ (250℃-30min)	0.75 ± 0.09	0.85 ± 0.03	0.60 ± 0.06	0.25 ± 0.06
$Cs_2AgBiBr_6$ (300℃-5min)	0.63 ± 0.15	0.85 ± 0.03	0.61 ± 0.05	0.17 ± 0.07
$Cs_2AgBiBr_6$ (300℃-15min)	0.82 ± 0.05	0.89 ± 0.01	0.53 ± 0.03	0.40 ± 0.03
$Cs_2AgBiBr_6$ (300℃-30min)	0.67 ± 0.08	0.87 ± 0.02	0.53 ± 0.03	0.31 ± 0.05

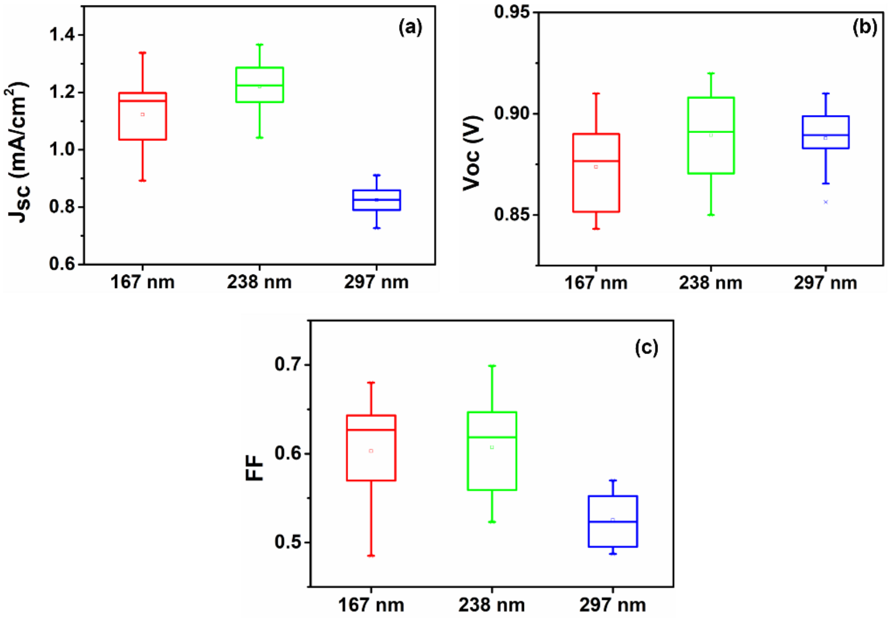

Fig. S9. The statistical box charts of open-circuit voltage (V_{oc}), short-circuit current density (J_{sc}) and fill factor (*FF*) of solar cells based on $Cs_2AgBiBr_6$ films with various thin film thickness. The values were obtained from 16 individual devices per annealing condition.

Table S2. Parameters of solar cell devices with different $Cs_2AgBiBr_6$ film thicknesses.

Sample	Jsc (mA/cm^2)	Voc (V)	FF	PCE (%)
$Cs_2AgBiBr_6$ (167 nm)	1.12 ± 0.12	0.87 ± 0.02	0.60 ± 0.06	0.53 ± 0.10
$Cs_2AgBiBr_6$ (238 nm)	1.22 ± 0.08	0.89 ± 0.02	0.61 ± 0.05	0.60 ± 0.05
$Cs_2AgBiBr_6$ (297 nm)	0.82 ± 0.05	0.89 ± 0.01	0.53 ± 0.03	0.40 ± 0.03

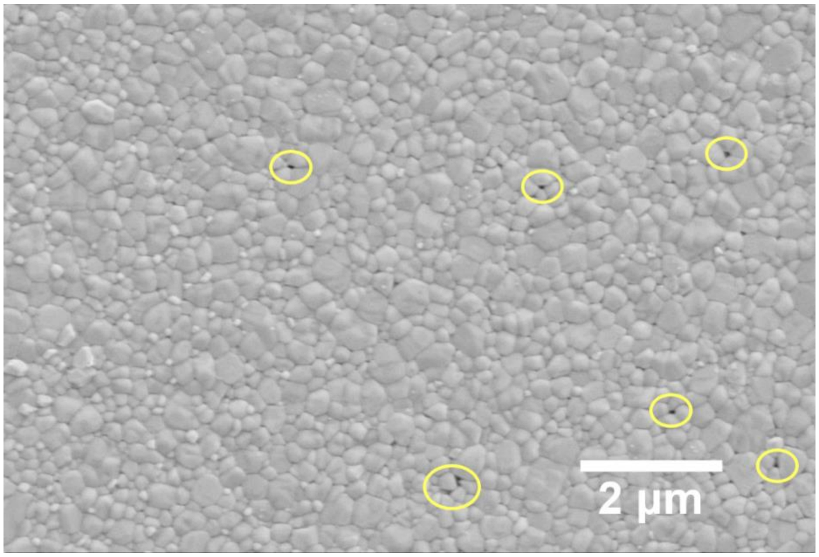

Fig. S10. SEM surface morphology of $Cs_2AgBiBr_6$ film annealed at 300 ℃ for 15 min. The film thickness is approximately 167 nm. The areas marked by yellow circles indicate pinholes in the $Cs_2AgBiBr_6$ film.

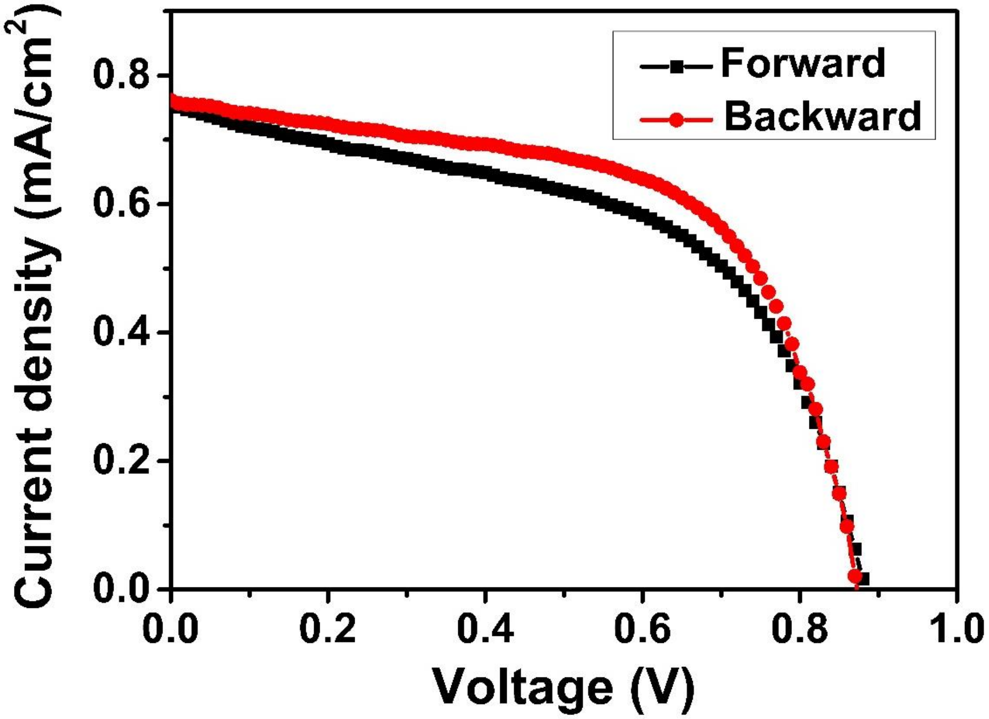

Fig. S11. J-V curves of $Cs_2AgBiBr_6$ solar cell, measured by backward scan and forward scan. The $Cs_2AgBiBr_6$ film was prepared at 300 ℃ for 30 min.
